# Orthostatic hypotension and cerebral small vessel disease: A systematic review

**DOI:** 10.1177/0271678X241283226

**Published:** 2024-09-16

**Authors:** Julia HI Wiersinga, Frank J Wolters, Mike JL Peters, Hanneke FM Rhodius-Meester, Marijke C Trappenburg, Majon Muller

**Affiliations:** 1 Amsterdam UMC location Vrije Universiteit Amsterdam, Department of Internal Medicine Section Geriatrics, Amsterdam, The Netherlands; 2 Amsterdam Cardiovascular Sciences, Atherosclerosis & Ischemic Syndromes, Amsterdam, The Netherlands; 3 Erasmus Medical Center, Department of Epidemiology, Rotterdam, The Netherlands; 4 Erasmus Medical Center, Departments of Radiology & Nuclear Medicine and Alzheimer Center Erasmus MC, Rotterdam, The Netherlands; 5 UMC Utrecht, University of Utrecht, Department of Internal Medicine Section Geriatrics, Utrecht, The Netherlands; 6 Oslo University Hospital, Department of Geriatric Medicine 155272 , Ullevål, Oslo, Norway; 7 Alzheimer Center Amsterdam, Department of Neurology, Amsterdam UMC, Vrije Universiteit Amsterdam, Amsterdam Neuroscience, Amsterdam, The Netherlands; 8 Amstelland Hospital, Department of Internal Medicine section Geriatrics, Amstelveen, The Netherlands

**Keywords:** BP (blood pressure), CSVD (cerebral small vessel disease), hypertension, OH (orthostatic hypotension), systematic review

## Abstract

Orthostatic hypotension(OH) is highly prevalent in ageing populations and may contribute to cognitive decline through cerebral small vessel disease(CSVD). Research on the association between OH and CSVD is fragmented and inconsistent. We systematically reviewed the literature for studies assessing the association between OH and CSVD, published until December 1st 2023 in MEDLINE, PubMed or Web of Science. We included studies with populations aged ≥60, that assessed OH in relation to CSVD including white matter hyperintensities(WMH), lacunes and cerebral microbleeds. Modified JBI checklist was used to assess risk of bias. A narrative synthesis of the results was presented. Of 3180 identified studies, eighteen were included. Fifteen studies reported on WMH, four on lacunes, seven on microbleeds. Six of fifteen studies on WMH found that OH was related to an increased burden of WMH, neither longitudinal studies found associations with WMH progression. Findings were inconsistent across studies concerning lacunes and microbleeds. Across outcomes, adequate adjustment for systolic blood pressure tended to coincide with smaller effect estimates. Current evidence on the OH-CSVD association originates mostly from cross-sectional studies, providing inconsistent and inconclusive results. Longitudinal studies using standardized and fine-grained assessment of OH and CSVD and adequate adjustment for supine blood pressure are warranted.

## Introduction

Orthostatic hypotension (OH) is a common condition in older adults, with a prevalence of approximately 10–30% in the older population.^1–4^ OH is defined as a blood pressure drop of at least 20 mmHg in systolic blood pressure (SBP) and/or 10 mmHg in diastolic blood pressure (DBP) within 3 minutes after standing up.^5^ OH is associated with risk of cardiovascular disease and stroke, as well as cognitive decline and dementia,^3,6^ but underlying mechanisms are uncertain. It has been hypothesized that the link between OH and dementia is due at least in part to the development of cerebral small vessel disease (CSVD) with episodic cerebral hypoperfusion. Several studies have reported on the association between OH and CSVD, but evidence is fragmented, with individual studies often too small to detect meaningful effects in varying markers of CSVD.

CSVD can be assessed on brain imaging as white matter hyperintensities, lacunes, perivascular spaces or microbleeds.^7^ The prevalence of CSVD increases with age, from about 5% at age 50 years to almost 100% among people older than 90 years.^8^ Cardiovascular disease and cardiovascular risk factors, such as high blood pressure, smoking, hypocholesteremia and diabetes contribute to the development of CSVD.^9,10^ OH might be related to CSVD through several mechanisms, including cerebral hypoperfusion, shared etiological mechanisms, or autonomous nervous system failure.^11,12^ First, OH occurs rapidly after standing up, which limits the potential for autoregulation to buffer these sudden BP changes, especially when vasoreactivity falters with larger blood pressure drops, resulting in cerebral hypoperfusion and oxidative stress. Oxidative stress in these vessels may contribute to microvascular dysfunction and damage, and ultimately leads to CSVD. Second, the relation between OH and CSVD may in part be accounted for by shared risk factors, with most importantly high systolic blood pressure (SBP), which is strongly associated with both OH and the development of CSVD.^13,14^ In patients with uncontrolled hypertension, OH is common and causes physicians to be reluctant with antihypertensive treatment to avoid symptomatic OH and falls. Even though scientific evidence suggests that treatment of hypertension is beneficial in patients with vascular OH, undertreatment of hypertension is common, and could results in associations between OH and CSVD if SBP is not properly accounted for.^15^ Third, cerebral damage caused by CSVD could lead to autonomous failure and OH (i.e., reverse causation). These mechanisms could be disentangled at least in part through longitudinal study of OH in relation to CSVD risk, accounting for shared etiological factors.

The aim of this study is to systematically review the existing literature on the association between OH and CSVD, taking into account study characteristics (which includes study population, study design), OH and CSVD measurement method, and possible confounding factors.

## Methods

This systematic review was reported in accordance with the Preferred Reporting Items for Systematic Reviews and Meta-Analyses (PRISMA) guidelines^16^ and registered with the PROSPERO International prospective register of systematic reviews [CDR number: 42022313278].

## Search strategy

To identify relevant publications, we conducted systematic searches in the bibliographic databases PubMed, Embase and Web of Science (Core Collection) from inception to December 1st 2023, in collaboration with a medical information specialist (RV). The following terms were used (including synonyms and closely related words) as index terms or free-text words: “Orthostatic hypotension”, “Cerebral small vessel disease”, “Cerebrovascular disease”. The references of the identified articles were searched for other studies meeting the criteria. All languages were accepted. Duplicate articles were excluded by a medical information specialist using Endnote X20.0.1 (Clarivatetm), following the Amsterdam Efficient Deduplication (AED)-method^17^ and the Bramer-method.^18^ The full search strategies for all databases can be found in Appendix A.

## Selection strategy

Two reviewers (JW and NS) independently screened all potentially relevant titles and abstracts using Rayyan^19^ for relevant, peer-reviewed original research articles published in the English language. The full text article was checked for the eligibility criteria if title and abstracts were not sufficient. Differences in judgement were resolved through consensus discussion. Inclusion criteria were: mean age >60 years; assessment of OH in accordance with the 1996 and 2011 consensus definition (i.e., drop in SBP >= 20 mmHg and/or DBP > = 10 mmHg within 3 min of standing);^5,20^ and assessment of CSVD, including either white matter hyperintensities, lacunes or microbleeds. We included qualitative, visual ratings of imaging markers, as well as quantitative, volumetric assessment of MRI. Studies without primary data, such as reviews, case reports and editorials, were excluded.

## Data extraction

The full text of the selected articles were assessed by two reviewers for full review and data extraction for:

- Study characteristics: author, year of publication, study design, study population and characteristics (mean age, sex, presence of orthostatic hypotension), in- and exclusion criteria.
- OH measurement method: active standing or tilt table test, duration of supine position, timing of blood pressure measurement, assessor of blood pressure measurement and further division of OH measurement.
- Measurement methods of main CSVD outcomes variables: white matter hyperintensities, lacunes and microbleeds (MRI sequence, scoring tools).
- Measure of association: odds ratio (OR), hazard ratio (HR), beta (β) or prevalence of outcome variables in patients with and without OH (including t-test statistics). If possible, OR’s were calculated from absolute numbers.
- Adjustment for confounding factors: demographic and clinical factors including, but not limited to, age, sex, systolic blood pressure, cardiovascular disease, diabetes, neurological disease and OH-inducing medication.

## Risk of bias assessment

Two reviewers (JW and HD) independently evaluated the methodological quality of the full text papers using the (modified) Johanna Briggs Institute (JBI) checklist for cross-sectional studies,^21^ comprising eight questions regarding risk of bias. We modified the generic questions to reflect the specific risks of bias regarding our research question (supplemental table 1). Any discrepancies were resolved through consensus discussion. Total risk of bias score was low if all items were scored low, moderate if one or two items were moderate/unclear or one item high, and high if more than one item high or more than two items moderate/unclear. Traffic light summary risk-of-bias plots for non-randomized included studies were produced by the risk-of-bias visualization (robvis) online tool to show risk of bias per JBI checklist question (D1-D8).^22^

## Analysis

We conducted a narrative synthesis of included studies and outcomes. Meta-analysis was planned for all outcomes, if a sufficient number of studies (i.e. at least three) reported on comparable exposure and outcome definitions in similar patient populations. After appraisal of all evidence, we decided against meta-analysis because of the large variety between studies. For example, measurement methods for OH varied substantially, as did measurement methods for CSVD. Moreover, reported measures of association were inconsistent. As such, for none of the outcomes there were more than three sufficiently comparable studies suitable for meta-analysis.

## Results

### Search results

**Figure 1.**
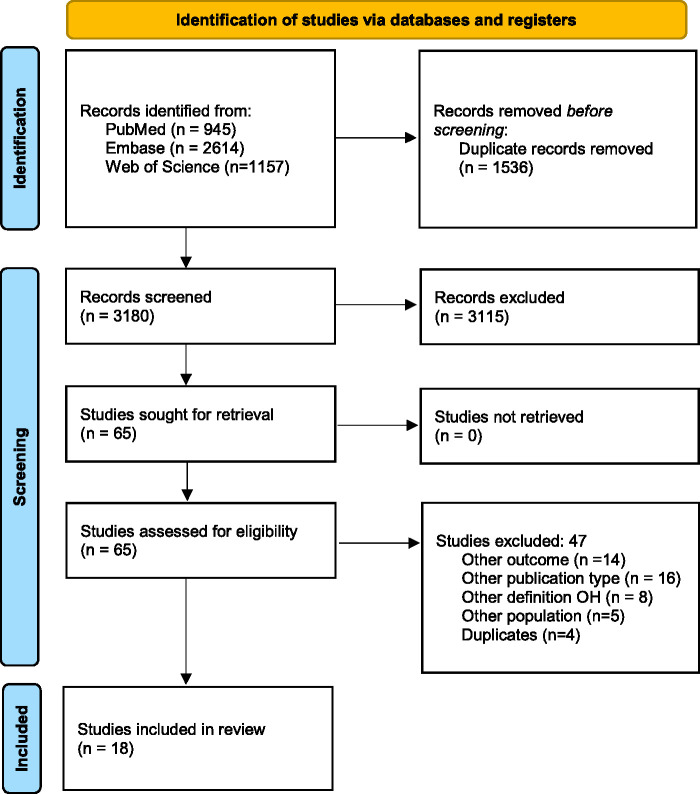
Selection process shown through PRISMA 2020 flow diagram.

The literature search generated a total of 4716 references: 945 in PubMed, 2614 in Embase and 1157 in Web of Science. After removing duplicates, 3180 unique references remained. The flow diagram of the search and selection process according to the PRISMA guidelines is presented in Figure 1. After title and abstract screening, 64 articles remained for full text screening, of which 18 were deemed eligible and were included.

### Summary of findings

Table 2 provides an overview of all included studies and their main results. The findings are stratified by CSVD outcome measure; white matter hyperintensities (Table 1), lacunes (Table 2) and microbleeds (Table 3).

**Table 1.** Summary of findings: Association orthostatic hypotension and white matter hyperintensities.

Author, year	Study design	Participants	OH assessment	Outcome assessment	Statistical adjustment	Outcomes
Buckley et al., 2020^23^	Cross-sectional cohort	N = 440 Irish community dwelling older adults.Mean age (yrs): 72 (range 65–92)51.5% female68.4% with OH	Continuous BP measurement for 2 minutes during active standing after 10 minutes supine rest.	**Imaging:** T3 MRI scanner, T2 and FLAIR images. **Assessor:** Radiologist, blinded to OH measurement.**WMH:** Scheltens score (0–84) converted in ordinal scale 0–10, 10–20, 20–30, >30.	**Adjusted for:** Age, sex, smoking, hypertension, hyperlipidemia, diabetes	Statistically significant association between OH and WMH at multiple time points.**Increase ordinal Scheltens score, different timepoints: OR (95%CI)**30 sec: 1.87 (1.11–3.14)70 sec: 2.50 (1.40–4.46)90 sec: 2.15 (1.15–3.99)110 sec: 2.48 (1.36–4.51)
Cui et al., 2020^24^	Cross-sectional cohort	N = 663 Chinese participants (aged >=60) presenting at community centers.Mean ± SD age (yrs): NOH: 68.1 ± 5.5, 1HOH: 69.1 ± 6.1, 2HOH: 69.2 ± 5.356.5% female32.3% one episode of OH14.6% two episodes of OH	Home measured OH (HOH), 3 consecutive days twice a day (morning before medication and after dinner). Measured in sitting position after 5 minutes, and 1 minute after standing up. Divided into 1 episode of HOH (1HOH), or ≥2 episodes of HOH (2HOH).	**Imaging:** 3 T MRI scanner, T2 and FLAIR images.**Assessor:** Experienced neuroradiologists, blinded to OH measurement.**WMH:** WMH volume (mL) with freesurfer programme, ratio WMH volume/total intracranial volume (TIV), Fazekas score, Fazekas score ≥2.	**Adjusted for:** Age, home SBP, smoking, LDL level, FPG, antihypertensive medication, anti-diabetes medication	Statistically significant association between OH and WMH.**Fazekas score ≥2 (OR, 95%CI):**1HOH : 2.12 (1.27–3.56) 2HOH : 4.91 (2.74–8.79)**White matter volume (mL), beta (95%CI):**1HOH : 0.73 (0.54–0.92) 2HOH : 1.54 (1.29–1.78)**WMH/TIV ratio (%), beta (95%CI):**1HOH : 0.05 (0.04–0.06)2HOH : 0.10 (0.09–0.12)
Foster-Dingley et al., 2018^34^	Cross-sectional cohort study (baseline data from randomized controlled trial)	N = 214 Older participants (+75) from DANTE study, Leiden, the Netherlands, with mild cognitive impairment using antihypertensive medication. Without serious cardiovascular disease or dementia.Mean ± SD age (yrs): 81 ± 4.157% female49% with OH.	Five minutes seated, blood pressure measurement at 1 and 3 minutes after standing up.	**Imaging:** 3 T MRI scan, T2 and FLAIR images**Assessor:** Volume with FMRIB Software Version 5.0.1. Library. Manual quality control check.**WMH:** WMH volume (mL) in automated manner	**Adjusted for:** Age and sex.	No statistically significant association between OH and WMH.**White matter volume (mL), beta (p-value):**−0.06 (p-value 0.37).
Huang et al., 2023^36^	Cross-sectional cohort	N = 211 patients with acute ischemic stroke attending First People’s Hospital of Huaihua, China.Mean ± SD age (yrs): 62.8 ± 10.834.5% female35.5% with OH.	Five minutes supine, blood pressure measurement at 1 and 3 minutes after standing up.	**Imaging:** Unclear**Assessor:** Two independent radiologists, blinded to the clinical history.**WMH:** Fazekas scale	**Adjusted for:** age, history of hypertension, history of CAD, hypotensive drugs	Statistically significant association between OH and WMH **Increase in Fazekas scale (OR, 95%CI):**1.29 (1.04–1.60)
Kario et al., 2002^37^	Cross-sectional cohort	N = 241 elderly hypertensive patients from participating clinics, Tochigi, Japan. Without renal failure, hepatic damage, coronary artery disease, stroke, symptomatic OH with SBP >30mmhg, diabetes mellitus.Mean ± SD age (yrs)NOH: 71 ± 6.5OH: 72 ± 6.256% female9.5% with OH	Tilt table test, supine position 10 minutes, OH measured between 6–10 minutes after tilting.	**Imaging:** 1.5 T MRI scan, T2 and FLAIR images.**Assessor:** Unclear, blinded to clinical history.**WMH:** Advanced deep WM lesion were defined as a detection of hyperintense multiple punctate lesions or early confluent stage or confluency.	**Not adjusted**	No statistically significant association between OH and WMH.**Prevalence deep white matter lesions (%):**NOH 31%OH 35%
Oh et al., 2013^29^	Cross-sectional cohort	N = 117 newly diagnosed Parkinson Patients from Seouls St. Mary’s Hospital, Seoul, Korea.Mean ± SD age (yrs):NOH 67.7 ± 11.1 OH 69.8 ± 8.061% female56% with OH	Tilt table test, supine position 30 minutes, OH measured between 2–5 minutes after tilting.	**Imaging:** 3 T MRI scanner, T2 and FLAIR images.**Assessor:** Unclear**WMH:** Scheltens Score (0–90).	**Adjusted for (ANCOVA):** age, history of hypertension, dm, LDL, serum homocysteine levels.	Statistically significant association between OH and WMH.**Total Scheltens score, p-value:**NOH 11.0 (8.2)OH 15.6 (9.6), p-value 0.017
Pilleri et al., 2013^30^	Cross-sectional matched cohort (matched on OH)	N = 48 matched participants from 108 consecutive subjects from IRCCS San Camillo PD Unit (Venice, Italy) diagnosed with Parkinson.Mean ± SD age (yrs):NOH: 65.6 ± 8.7OH: 64.9 ± 9.754% female48% with OH (matched participants).	Tilt table test, supine position 10 minutes, OH measured every minute from 1 to 3 minutes after tilting.	**Imaging:** 1.5 T MRI, T2 and FLAIR images.**Assessor:** Independently performed by two trained physicians, blinded about patients’ clinical state**WMH:** Scheltens Score (0–84).	**Not adjusted**	No statistically significant association between OH and WMH.**Total Scheltens score, p-value:**NOH 10.6 ± 7.5OH 10.3 ± 8.3, p-value 0.57
Pilotto et al., 2021^31^	Cross-sectional cohort	N = 384 patients from imaging repositories diagnosed with Parkinson or Dementia Lewy Bodies constituted by 8 specialized Movement Disorder and Dementia Centers in the United States, Canada, Italy and Japan. Patients without neurogenic OH, comorbid neuropathy, conditions associated with cognitive deficits, antihypertensive or antipsychotic drugs, history of CVA/TIA, history of drug of alcohol abuse.Mean ± SD age (yrs): 68.4 ± 11.136.9 % female44.3 % with OH	5 minutes of rest, blood pressure measurement within 3 minutes after standing up.	**Imaging:** Clinical imaging repository, T1- and T2 weighted images.**Assessor:** Analyzed in a centralized fashion by 4 independent raters.**WMH:** Scheltens scale for 4 regions (and per hemisphere). 0 = no WMH, 1= punctiform, 2 = early confluent, 3 = confluent. All added up.	**Adjusted for (ANCOVA):** age, sex, education, diagnosis and disease duration.	No statistically significant association between OH and WMH.**Total Scheltens scale, p-value:**NOH 5.5 ± 5.3OH 5.5 ± 5.5, p-value 0.49OH+SH- : 5.2OH+SH+ : 6.5, p-value 0.72
Shin et al., 2021^33^	Cross-sectional cohort	N = 154 patients with Parkinson Disease with MRI and clinical assessment from Seouls St. Mary’s Hospital, Seoul, Korea.Mean ± SD age (yrs): 70.2 ± 9.048.7% female19.4% with OH	Tilt table test, supine position for 20 minutes, blood pressure measurement 3 or 5 minutes after tilting.	**Imaging:** 3 T MRI, FLAIR images.**Assessor:** neuroradiologist, blinded to clinical data.**WMH:** Periventricular and deep WMH both scored on Fazekas scale, total score 0–6.	**Adjusted for:** age, sex, disease duration, levodopa-equivalent dose, smell identification test score, depression.	No statistically significant association between OH and WMH.**White matter grade (0–6), beta, p-value:**−0.07 (0.29)
Soennesyn et al., 2012^28^	Cross-sectional cohort	N = 139 patients with mild dementia from dementia clinics in Norway. Mean (IQR) age (yrs): 76.9 (I71–81)57.0% female26.0% with OH	In supine position (duration unclear), blood pressure measurement within 3 minutes after standing up.	**Imaging:** 1.5 T MRI, FLAIR, T1 en T2 images.**Assessor:** Volumetric assessment with method developed/published by Firbank et al. Manually checked by physician.Visual assessment by experienced rater, blind to clinical data.**WMH:** *Semi quantative assessment*: Scheltens scale.*Volumetric assessment* (n = 82): White matter volume and WMH/TIV ratio. Both assessments divided in quintiles and comparison between lowest and highest quintile.	**Adjusted for:** age, sex, AD, history of hypertension, diabetes, coronary heart disease, heart failure, stroke APOEe4 allele, smoker.	No statistically significant association between OH and WMH.**OH fractions in lowest and highest quintile (p-value):**Semi quantative group:Lowest quintile 12/25, highest quintile 14/28 (p = 1.00)Volumetric group: Lowest quintile 10/17, highest quintile 10/19 (p = 0.97)**Logistic and multinomial regression:** no significant association (data not shown).
Ten Harmsen et al., 2018^32^	Cross-sectional cohort	N = 204 patients with Parkinson Disease evaluated at Radboud UMC, The Netherlands. Mean ± SD age (yrs): 64.6 ± 10.869.1% female26.0% with OH	In prospective cohorts (n = 78) OH was based on measurements in supine position (duration unclear), and blood pressure measurement 3 minutes after standing up.In retrospective cohort based on complaints of OH.	**Imaging:** 3 T MRI, FLAIR and T2 images.**Assessor:** Consistently by the same neuroradiologist under supervision.**WMH:** Periventricular and deep WMH both scored on Fazekas scale (0–3).	**Adjusted for:** age, sex, dopaminergic medicatie use, hypertension, hoehn & yahr stadium, cognitive decline.	Statistically significant association between OH and WMH.**Fazekas score ≥ 2, (%, p-value), entire cohort:** Periventricular WML : NOH: 25%, OH: 40% (p = 0.34)Deep WML:NOH: 15%, OH: 30% (p = 0.03)**WMH, odds ratio (95CI%), entire cohort:**Periventricular WML: 0.70 (0.32–1.43), Deep WML: 0.41 (0.18–0.92)
Wiersinga et al., 2023^35^	Cross-sectional cohort	N = 3971 patients visiting memory clinic in Amsterdam UMC, The Netherlands, from the Amsterdam Aging Cohort, no specific diagnosis.Mean ± SD age (yrs): 67.5 ± 10.844.9% Female9.5% with EOH18.2% with DPOH	Supine 3 minutes, OH after 1 and 3 minutes, divide in early OH (only at 1 minute) and delayed/prolonged OH (at 1 and 3 or only at 3 minutes).	**Imaging:** CT or 1.5 T MRI, FLAIR and T2 images.**Assessor:** Two trained specialists and supervised by a clinical radiologist according to the local dementia protocol.**WMH:** Fazekas score ≥ 2	**Adjusted for:** age, sex, OH-inducing medication, MMSE score, CVD, diabetes and supine SBP.	No statistically significant association between OH and WMH**Fazekas score ≥ 2 (OR, 95%CI),**EOH: 0.95 (0.73–1.23)DPOH: 1.04 (0.85–1.27)
Zimmerman et al., 2020^27^	Cross-sectional cohort	N = 495 patients from the TREND-study, including elderly patients without neurodegenerative diseases, in Tübingen, Germany. 93 patients with MRI data.Mean age (yrs): NOH 63.9, OH 62.946.9% female17.6% with OH	Supine (duration unclear) and blood pressure measurement at 30/90/150/210 sec of active standing, OH at any time point.	**Imaging:** 3 T MRI, T2 images.**Assessor:** Neurologist trained in the interpretation of MRI scans. **WMH:** Fazekas score (0–3).	**Not adjusted**	Borderline statistically significant association between OH and WMH.**Fazekas score ≥ 1, (%, p-value):**NOH: 68.8%, OH: 91.7% (p = 0.09)
Longitudinal studies			
Jacob et al., 2021^25^	Cross-sectional and longitudinal cohort	N = 503 Community dwelling older adults from RUN-DMC study, Radboud University Nijmegen, The Netherlands, undergoing MRI. Aged between 50–80, visible SVD on MRI.Follow-up inclusion:Baseline with MRI: n = 503Re-examined at 5 years with MRI: n = 361Re-examined at 9 years with MRI: n = 296Mean ± SD age (yrs): 65.5 ± 8.856.5% female9.1% with OH.	Five minutes supine, blood pressure measurement at 1 minute after standing up.	**Imaging:** 1.5 T MRI, T2 and flair imaging**Assessor:** semi-automatic WMH segmentation. Visually checked by one trained rater, blinded for clinical data.**WMH:** WMH volumes (mL) using semi autonomic WMH segmentation	**Adjusted for:** age, sex, hypertension, OH inducing drugs.	Significant association between OH and WMH cross sectional. **White matter volume (mL), beta (95%CI):**0.18 (0.02–0.34)No statistically significant association between OH and WMH longitudinally **WMH progression, OR (95%CI)**3.63 (0.29–6.98)
Juraschek et al., 2024^15^	Cross-sectional and longitudinal cohort	N = 3147 community based older (65+) participants of the cardiovascular health study in the United States of America. Without dementia or stroke. Follow-up inclusion:Baseline with MRI: n = 3147Re-examined at 5 years with MRI: n = 1858Mean ± SD age (yrs): 72.7 ± 5.557.6% female15.9% with OH.	OH measured after supine rest of 20–30 minutes and 30 seconds of seating, measured 3 minutes after standing up.	**Imaging:** 1.5 T or 3 T MRI scan, T2 and flair images**Assessor:** Two trained readers using the same CHS protocol. Unaware of initial or follow-up scan.**WMH:** White matter grade (0–9), not further specified.	**Adjusted for:** age, sex, race, research clinic, BMI, HDL, triglycerides, diabetes, hypertension, SBP, DBP, diuretic use, beta blocker use, education, current drinking status, smoking.	No statistically significant association between OH and WMH.**White matter grade, beta (95%CI):**0.01 (−0.12–0.14)**White matter worsening (change in grade), (95%CI):** 0.04 (−0.10–0.19)

BMI: body mass index; BP: blood pressure; CAD: coronary artery disease; CI: confidence interval; CT: computer tomography; CVD: cardiovascular disease; DBP: diastolic blood pressure; FLAIR: fluid-attenuated inversion recovery; FPG: fasting plasma glucose; HDL: high density lipoprotein; IQR: interquartile range; LDL: low density lipoprotein; mL: milliliter; MMSE: mini-mental state exam; MRI: magnetic resonance imaging; N: number; NOH: no orthostatic hypotension; OH: orthostatic hypotension; OR: odds ratio; SBP: systolic blood pressure; SD: standard deviation; TIV: total intracranial volume; WMH: white matter hyperintensities; WML: white matter lesions; yrs: years.

**Table 2.** Summary of findings: Association orthostatic hypotension and lacunes.

Author, year	Study design	Participants	OH assessment	Outcome assessment	Statistical adjustment	Outcomes
Cui et al., 2020^24^	Cross-sectional cohort	N = 663 Chinese participants (aged >=60) presenting at community centers.Mean ± SD age (yrs): NOH: 68.1 ± 5.5,1HOH: 69.1 ± 6.1, 2HOH: 69.2 ± 5.356.5% female32.3% one episode of OH14.6% two episodes of OH	Home measured OH (HOH), 3 consecutive days twice a day (morning before medication and after dinner). Measured in sitting position after 5 minutes, and 1 minute after standing up. Divided into 1 episode of HOH (1HOH), or ≥2 episodes of HOH (2HOH).	**Imaging:** 3 T MRI scanner, T2 and FLAIR images.**Assessor**: Experienced neuroradiologists, blinded to OH measurement.**Lacunes:** Presence of lacunes (≥1)	**Adjusted for**: Age, home SBP, smoking, LDL level, FPG, antihypertensive medication, anti-diabetes medication.	Statistically significant association between 2HOH and lacunes. **Lacunes ≥1 (OR, 95%CI)**:1HOH 1.89 (0.89–3.08), 2HOH 5.36 (2.99–9.61)
Foster-Dingley et al., 2018^34^	Cross-sectional cohort (baseline data from randomized controlled trial)	N = 214 Older participants (+75) from DANTE study, Leiden, the Netherlands, with mild cognitive impairment using antihypertensive medication. Without serious cardiovascular disease or dementia. Mean ± SD age (yrs): 81 ± 4.157% female49% with OH.	Five minutes seated, blood pressure measurement at 1 and 3 minutes after standing up.	**Imaging**: 3 T MRI scan, T2 and FLAIR images**Assessor**: Assessor unclear. All measurements were obtained blinded to participants’ demographic and clinical information.**Lacunes**: Presence of lacunes (≥1)	**Adjusted for:** Age, sex	No statistically significant association between OH and lacunes.**Lacunes ≥ 1, OR (95%CI)**:1.16 (0.65–2.08)
Jacob et al., 2021^25^	Cross-sectional cohort	N = 503 Community dwelling older adults from RUN-DMC study, Radboud University Nijmegen, The Netherlands, undergoing MRI. Aged between 50–80, visible SVD on MRI.Mean ± SD age (yrs): 65.5 ± 8.856.5% female9.1% with OH.	Five minutes supine, blood pressure measurement at 1 minute after standing up.	**Imaging**: 1.5 T MRI, T2 and flair imaging**Assessor:** Manually by two trained raters, blinded for clinical data. **Lacunes**: Presence of lacunes (≥1)	**Adjusted for**: age, sex, hypertension, hypercholesterolemia, OH inducing drugs.	No statistically significant association between OH and lacunes.**Lacunes ≥1, OR (95%CI)**:1.88 (0.97–3.61)
Wiersinga et al., 2023^35^	Cross-sectional cohort	N = 3971 patients visiting memory clinic in Amsterdam UMC, The Netherlands, from the Amsterdam Aging Cohort, no specific diagnosis.Mean ± SD age (yrs): 67.5 ± 10.844.9% Female9.5% with EOH18.2% with DPOH	Supine 3 minutes, OH after 1 and 3 minutes, divide in early OH (only at 1 minute) and delayed/prolonged OH (at 1 and 3 or only at 3 minutes).	**Imaging:** CT or 1.5 T MRI, FLAIR and T2 images.**Assessor**: Two trained specialists and supervised by a clinical radiologist according to the local dementia protocol.**Lacunes:** Presence of lacunes (≥1)	**Adjusted for**: age, sex, OH-inducing medication, MMSE score, CVD, diabetes and supine SBP.	No statistically significant association between OH and lacunes.**Lacunes ≥1 (OR, 95%CI),**EOH: 0.70 (0.47–1.06)DPOH: 1.21 (0.91–1.62)

BMI: body mass index; BP: Blood pressure; CAD: Coronary artery disease; CI: confidence interval; CT: computer tomography; CVD: cardiovascular disease; DBP: diastolic blood pressure; FLAIR: fluid-attenuated inversion recovery; FPG: fasting plasma glucose; HDL: high density lipoprotein; IQR: interquartile range; LDL: low density lipoprotein; MMSE: mini-mental state exam; MRI: magnetic resonance imaging; N: number; NOH: no orthostatic hypotension; OH: orthostatic hypotension; OR: odds ratio; SBP: systolic blood pressure; SD: standard deviation; yrs: years.

**Table 3.** Summary of findings: Association orthostatic hypotension and microbleeds.

Author, year	Study design	Participants	OH assessment	Outcome assessment	Statistical adjustment	Outcomes
Cui et al., 2020^24^	Cross-sectionalCohort	N = 663 Chinese participants (aged >=60) presenting at community centers.Mean ± SD age (yrs): NOH: 68.1 ± 5.5, 1HOH: 69.1 ± 6.1, 2HOH: 69.2 ± 5.356.5% female32.3% one episode of OH14.6% two episodes of OH	Home measured OH (HOH), 3 consecutive days twice a day (morning before medication and after dinner). Measured in sitting position after 5 minutes, and 1 minute after standing up. Divided into 1 episode of HOH (1HOH), or ≥2 episodes of HOH (2HOH).	**Imaging:** 3 T MRI scanner, T2 and FLAIR images.**Assessor**: Experienced neuroradiologists, blinded to OH measurement.**Microbleeds:** Total number of microbleeds and presence of microbleeds (≥1).	**Adjusted for**: Age, home SBP, smoking, LDL level, FPG, antihypertensive medication, anti-diabetes medication.	Statistically significant association between OH and microbleeds. **Microbleeds ≥1 (OR, 95%CI)**:1HOH 2.16 (1.27–3.69), 2HOH 3.05 (1.62–5.74)
Daida et al., 2018^41^	Cross-sectional cohort	N = 124 patients with Parkinson Disease admitted to Juntendo University Hospital, Tokyo, Japan, for diagnostic assessment, drug adjustment or deep-brain stimulation.Mean ± SD age (yrs): 63.6 ± 10.554.5% female47.6% with OH	Supine rest 15 minutes, blood pressure measurement within 3 minutes of standing up.	**Imaging**: 1.5 T MRI, FLAIR images.**Assessor**: Trained observer, blinded to patients’ clinical data.**Microbleeds**: Presence of microbleeds (≥1), divided into deep/infratentorial microbleeds and lobar microbleeds.	**Not adjusted**.	Statistically significant association between OH and microbleeds.**Microbleeds ≥1, (%, p-value)**:NOH: 6.2%, OH: 23.7%, p-value 0.01
Foster-Dingley et al., 2018^34^	Cross-sectionalCohort (baseline data from randomized controlled trial)	N = 214 Older participants (+75) from DANTE study, Leiden, the Netherlands, with mild cognitive impairment using antihypertensive medication. Without serious cardiovascular disease or dementia. Mean ± SD age (yrs): 81 ± 4.157% female49% with OH.	Five minutes seated, blood pressure measurement at 1 and 3 minutes after standing up.	**Imaging**: 3 T MRI scan, T2 and FLAIR images**Assessor**: Assessor unclear. All measurements were obtained blinded to participants’ demographic and clinical information.**Microbleeds**: Presence of microbleeds (≥1)	**Adjusted for:** Age, sex	No statistically significant association between OH and microbleeds.**Microbleeds ≥1, OR, (95%CI)**:0.94 (0.52–1.69)
Wiersinga et al., 2023^35^	Cross sectional cohort	N = 3971 patients visiting memory clinic in Amsterdam UMC, The Netherlands, from the Amsterdam Aging Cohort, no specific diagnosis.Mean ± SD age (yrs): 67.5 ± 10.844.9% Female9.5% with EOH18.2% with DPOH	Supine 3 minutes, OH after 1 and 3 minutes, divide in early OH (only at 1 minute) and delayed/prolonged OH (at 1 and 3 or only at 3 minutes).	**Imaging:** CT or 1.5 T MRI, FLAIR and T2 images.**Assessor**: Two trained specialists and supervised by a clinical radiologist according to the local dementia protocol**Microbleeds:** Presence of microbleeds (≥3)	**Adjusted for**: age, sex, OH-inducing medication, MMSE score, CVD, diabetes and supine systolic blood pressure.	No statistically significant association between OH and microbleeds.**Microbleeds ≥3 (OR, 95%CI):**EOH: 1.12 (0.74–1.67)DPOH: 0.95 (0.68–1.31)
Jacob et al., 2024^25^	Cross-sectional cohort	N = 503 Community dwelling older adults from RUN-DMC study, Radboud University Nijmegen, The Netherlands, undergoing MRI. Aged between 50–80, visible SVD on MRI.Mean ± SD age (yrs): 65.5 ± 8.856.5% female9.1% with OH.	Five minutes supine, blood pressure measurement at 1 minute after standing up.	**Imaging**: 1.5 T MRI, T2 and flair imaging**Assessor:** Manually by two trained raters, blinded for clinical data. **Microbleeds**: Presence of microbleeds (≥1)	**Adjusted for**: age, sex, hypertension, hypercholesterolemia, OH inducing drugs.	Statistically significant association between OH and microbleeds. **Microbleeds ≥1, OR (95%CI)**:2.37 (1.16–4.68)
Yamashiro et al., 2015^42^	Cross-sectional cohort	N = 167 patients with Parkinson Disease admitted to the Department of Neurology, Juntendo University Hospital, Tokyo, Japan.Mean ± SD age (yrs): 70.1 ± 7.956.0% female46.9% with OH	Supine/seating BP (duration unclear), pressure measurement within 3 minutes of standing up, only systolic blood pressure measurement used for OH. Only assessed when symptoms of OH were present.	**Imaging**: 1.5 T MRI, FLAIR images.**Assessor**: Trained observer blinded to patients’ clinical data.**Microbleeds**: Presence of microbleeds (≥1), divided into deep/infratentorial microbleeds and lobar microbleeds.	**Adjusted for**: Age, hypertension, ischemic stroke, antiplatelet use	No statistically significant association between OH and any microbleeds, statistically significant association between OH and deep/infratentorial microbleeds. **Any microbleeds >1 (OR, 95CI%):**2.38 (0.78–7.05)**Deep/infratentorial microbleeds >1 (OR, 95CI%)**:5.11 (1.57–17.5)
Yamashiro et al., 2018^43^	Cross-sectional cohort	N = 128 patients with Parkinson Disease recruited at department of Neurology, Juntendo University Hospital, Tokyo, Japan.Mean ± SD age (yrs): 64.4 ± 9.756.0% female46.9% with OH	Supine for 15 minutes, pressure measurement within 3 minutes of standing up.	**Imaging**: 3 T MRI, FLAIR images.**Assessor**: Trained observer blinded to patients’ clinical data. **Microbleeds**: Presence of microbleeds (≥1), divided into deep/infratentorial microbleeds and lobar microbleeds.	**Not adjusted**	No statistically significant association between OH and any microbleeds.**Any microbleeds, %:**NOH: 10%, OH: 12% (p > 0.05)**Deep/infratentorial microbleeds, %:**NOH: 3%, OH: 9% (p > 0.05)

BMI: body mass index; BP: Blood pressure; CAD: Coronary artery disease; CI: confidence interval; CT: computer tomography; CVD: cardiovascular disease; DBP: diastolic blood pressure; FLAIR: fluid-attenuated inversion recovery; FPG: fasting plasma glucose; HDL: high density lipoprotein; IQR: interquartile range; LDL: low density lipoprotein; MMSE: mini-mental state exam; MRI: magnetic resonance imaging; N: number; NOH: no orthostatic hypotension; OH: orthostatic hypotension; OR: odds ratio; SBP: systolic blood pressure; SD: standard deviation; SVD: small vessel disease; yrs: years.

#### Orthostatic hypotension and white matter hyperintensities

Fifteen studies reported on the association between OH and white matter hyperintensities, of which 13 were cross-sectional and 2 longitudinal by design. Six studies included community-dwelling older adults.^23–28^ The other nine studies were clinic-based, including patients with Parkinson’s Disease (5 studies),^29–33^ cognitive complaints (3 studies)^28,34,35^ or cardiovascular disease (2 studies).^36,37^ Sample sizes ranged from 48 patients with Parkinson’s disease to 5700 adults in the largest community-based study.^26,31^ Most studies (n = 10) used active standing for the assessment of OH,^23,25,27,28,31,32,34–36,38^ four used the tilt table test^29,30,33,37^ and one used home measured OH (measured after active standing).^24^ The time from standing up till repeated BP measurement varied, with some studies measuring at one and three minutes,^30,34,36^ whereas other measured only at one minute,^24,25^ only at three minutes,^26,29,32,33,35^ later than three minutes,^37^ or some unspecified moment within three minutes after standing up.^23,28,31^ The majority of studies used visual ratings of WMH as the outcome measures, notably the Fazekas score,^24,27,32,35,36,39^ or Scheltens score,^23,28–31,40^ or other visual ratings such as white matter grade, advanced deep white matter lesions, or adjusted Fazekas score.^24,26,33,37^ Four studies used volumetric assessment of white matter hyperintensities.^24,25,28,34^

Among all 13 cross-sectional studies, six found a significant association between OH and increased risk of having white matter hyperintensities,^23–25,29,32,36^ with effect size (OR, 95%CI) ranging from 1.29 (1.04–1.60) point increase in the Fazekas scale in patients with ischemic stroke to 4.91 (2.74–8.79) risk of Fazekas scale ≥2 in community dwelling older patients. Nine studies did not report a significant association,^26–28,30,31,33–35,37^ with mostly non-significant differences in Scheltens scores or white matter volume presented. The number of participants in these studies ranged between 48 PD patients to 3971 patients with cognitive complaints.

Among two longitudinal studies, one included 504 participants with CSVD, of whom 361 (72%) were re-examined after 5 years and 296 (59%) again after 9 years. The other study included 3147 community-dwelling individuals, of whom 1858 (59%) underwent repeated MRI after 5 years of follow-up. Neither of these 2 longitudinal studies found a statistically significant association between OH and risk of developing or progression of white matter hyperintensities (OR [95%CI]: 3.63 [0.29–6.98], and beta [95%CI]: 0.04 [−0.10–0.19], respectively).^25,26^

Thorough adjustment for potential confounders appeared associated with smaller effect estimates (Table 2). Notably, none of the studies that adjusted for SBP (as a continuous variable) reported a significant association;^25,26,35^ of studies adjusting for history of hypertension, four out of five had statistically significant results.^23,28,29,32,36^ With regard to means of OH assessment, five studies focused specifically on OH at three minutes after standing up, of which three found that OH was associated with increased risk of white matter hyperintensities.^29,32,35^ Five out of eight studies assessing OH at a time point shorter than three minutes after standing up observed no association with WMH.^26,33^ Kario et al assessed OH at 6–10 minutes after tilting, and found no association.^37^

**Figure 2.**
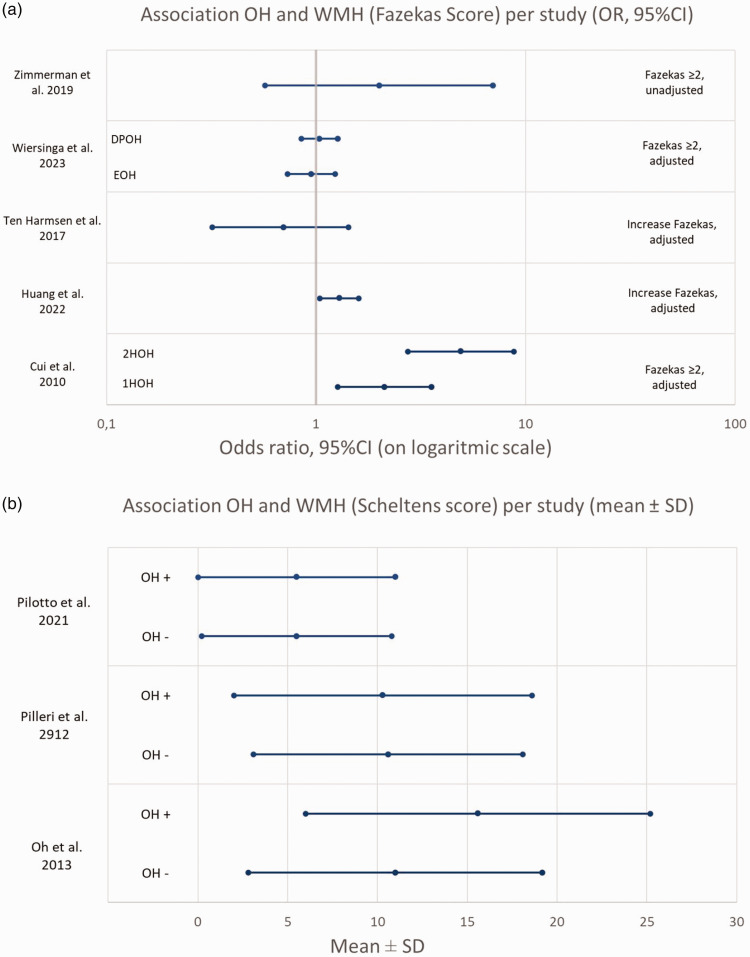
(a) Association OH and Fazekas score, OR’s (95%CI) per study (given or calculated) and (b) Association OH and Scheltens score, mean Scheltens score ± SD for patients with and without OH, per study.

To summarize the results, all OR’s (given or calculated from prevalence) for association between OH and WMH (Fazekas score), and mean ± SD for Scheltens scores were combined in Figure 2. It was not possible to include all studies due to the high heterogeneity between studies and no pooled estimate can be produced. Therefore the figure should be interpreted with caution.

#### Orthostatic hypotension and lacunes

Four cross-sectional studies reported on the association between OH and lacunes, performed in older patients with cognitive complaints visiting the memory clinic,^34,35^ older community dwelling older adults,^24^ or patients with SVD.^25^ All studies had a cross-sectional study design and used > = 1 lacunes as cut-off value. All these studies used active standing for the measurement of OH, with one study using home-measured OH (divided into measured once or more than once during the three day self-measurement period),^24^ and one study dividing OH in Early OH (only at 1 minute) and delayed/prolonged OH (only at 3, or at 1 and 3 minutes).^35^

One of four studies reported a significant association between OH and the presence of lacunes,^24^ whereas the other three studies did not.^25,34,35^ The study that found an association was performed in community dwelling older adults (OR [95%CI] for home-measured OH once: 1.89 [0.89–3.08], and for home measured OH more than once: 5.36 [2.99–9.61]). Both studies among memory clinic outpatients found no association between OH and lacunes: OR (95%CI) of 1.16 (0.65–2.08)^34^ and OR (95%CI) for early OH of 0.70 (0.47–1.06) and delayed/prolonged OH 1.21 (0.91–1.62)), respectively.^35^ In patients with SVD the OR (95%CI) was 1.88 (0.97–3.61).

**Figure 3.**
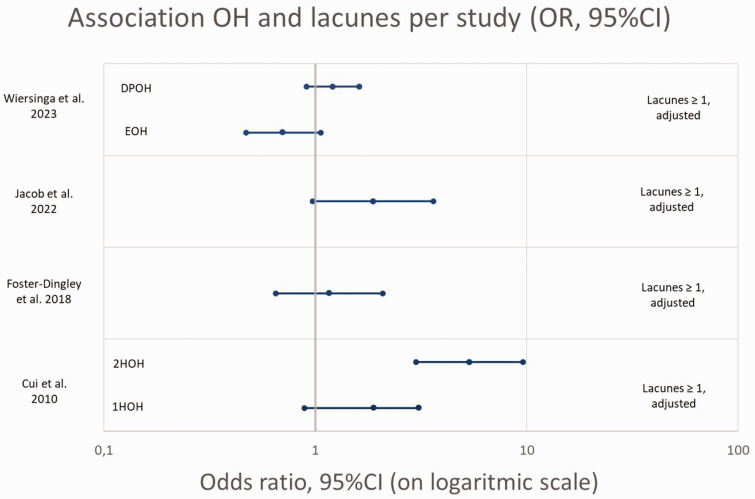
Association OH and presence of lacunes, OR’s (95%CI) per study (given or calculated).

All OR’s for association between OH and lacunes are presented in Figure 3. Due to the low number of studies and high heterogeneity between studies no pooled estimate could be produced and the figure should be interpreted with caution.

#### Orthostatic hypotension and microbleeds

Seven cross-sectional studies reported results on OH and microbleeds, of which three studies included patients with Parkinson’s disease,^41–43^ one study included community dwelling older adults,^24^ two included patients with cognitive complaints visiting a memory clinic,^34,35^ and one with patients with SVD.^25^ Six studies used ≥1 microbleeds as cut-off value whilst one study used ≥3 microbleeds.^35^ Two studies further divided presence of microbleeds into lobar microbleeds and deep/infratentorial microbleeds.^42,43^ All studies used active standing for the measurement of OH, one used HOH^24^ and one divided into early and delayed/prolonged OH.^35^ Furthermore one study only assessed OH when typical complaints of OH were present.^42^

Overall three studies found a significant association between OH and presence of microbleeds,^24,25,41^ and four studies did not.^34,35,42,43^ One of the two that segregated lobar from deep microbleeds showed an association between OH and deep/infratentorial microbleeds (OR (95%CI) 5.11(1.57–17.5)), but not significantly with lobar microbleeds (OR (95%CI) 2.38 (0.78–7.05)), adjusted for important confounders (age, sex, hypertension, history of CVD, diabetes), the other showed a prevalence of 3% in patients without OH, compared to 9% in OH patients (p-value >0.05). This study showed that specifically the combination of OH with supine hypertension was associated with the presence of microbleeds at any location, (OR (95%CI) 1.29 (1.04–1.60)),^43^ adjusted for hypertension, diabetes, history of stroke, antiplatelet treatment and WMH.

**Figure 4.**
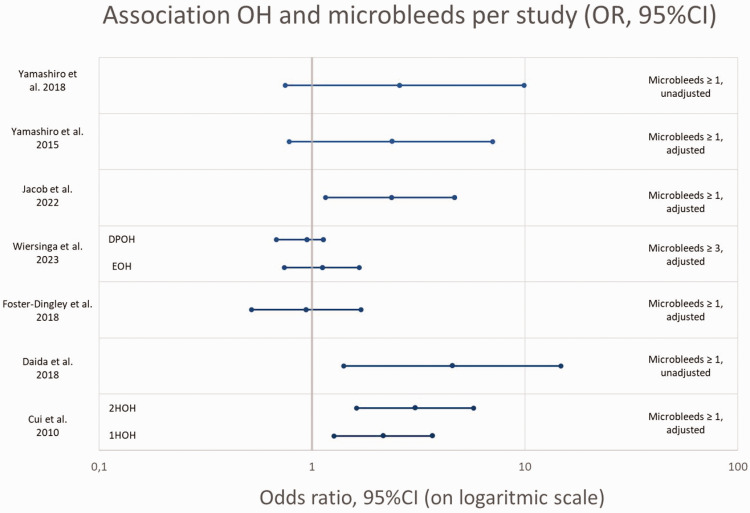
Association OH and presence of microbleeds, OR’s (95%CI) per study (given or calculated).

The OR’s for the association between OH and microbleeds (given or calculated) are presented in Figure 4. Due to the high heterogeneity between studies, in OH measurement method, microbleed definition, and adjustments for confounders, no pooled estimate could be produced and the figure should be interpreted with caution.

## Risk of bias assessment

**Figure 5.**
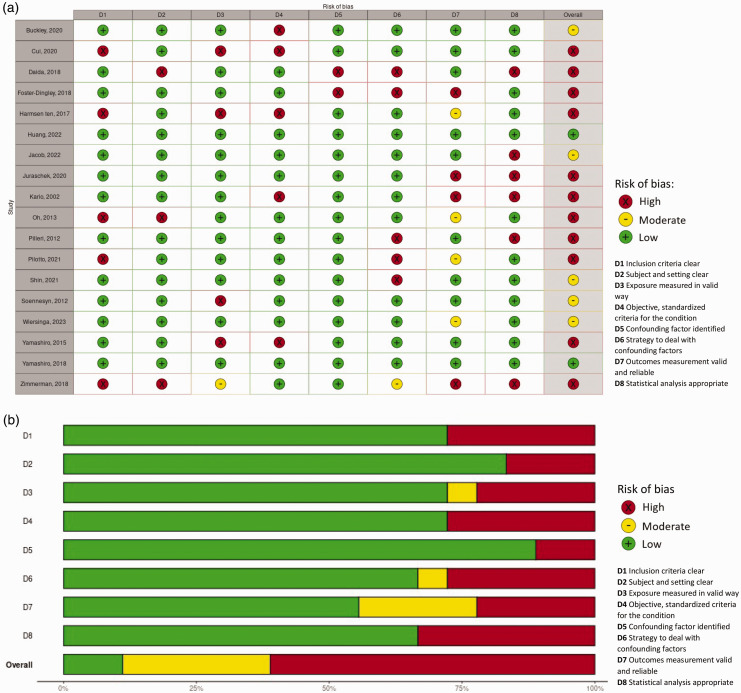
(a) Risk of bias traffic light plot. D1 Inclusion criteria clear, D2 Subjects and setting clear, D3 Exposure measured in valid way, D4 Objective, standardized criteria for the condition, D5 Confounding factor identified, D6 Strategy to deal with confounding factors, D7 Outcomes measurement valid and reliable, D8 Statistical analysis appropriate. Judgement: High, moderate, low and (b) Risk of bias summary plot (unweighted). D1 Inclusion criteria clear, D2 Subjects and setting clear, D3 Exposure measured in valid way, D4 Objective, standardized criteria for the condition, D5 Confounding factors identified, D6 Strategy to deal with confounding factors, D7 Outcomes measurement valid and reliable, D8 Statistical analysis appropriate. Judgement: High, moderate, low.

The risk of bias assessment is visualized in Figure 5. Overall risk of bias was low in two out of eighteen studies (11%), moderate in five studies (28%) and high in eleven studies (61%), mostly due to risk of bias in measurement of OH (D4, high risk in 28%), dealing with confounding factors (D6, high risk in 28%, moderate risk in 6%), measurement of CSVD (D7, high in 17%, moderate in 22%, largely due to the lack of (information on) blinding) or inappropriate statistical analysis (D8, high in 33%).

Supplementary figure 1 shows a visualized summary of findings per outcome measure, color coded for risk of bias (low - green, moderate - yellow, high - red) and the direction of the result (positive association - green and no/negative association - red).

## Meta-analysis

As stated before, no meta-analysis could be performed due to the large variety between studies.

## Discussion

In this systematic review of studies on the association between OH and CSVD in older adults, we included 16 cross-sectional and 2 longitudinal studies. Taken together, there was no consistent association between OH and white matter hyperintensities, lacunes or microbleeds. However, effect estimates varied substantially across studies, driven at least in part by heterogeneity in study populations and research methods.

There are several possible patterns in the results and possible factors explaining the differences in study outcome and therefore the inconclusive results. First, there was a large variability in study populations such as community-dwelling older adults, patients with Parkinson’s Disease, with memory complaints, or with dementia. The pathophysiology of OH in these groups might differ, with a larger contribution of autonomic dysfunction in patients with α-synucleopathy (i.e., Parkinson’s disease or Lewy body dementia) or more generally neurodegeneration in memory clinic populations, compared to predominantly hypertension or vascular disease related OH in population-based cohorts. In population-based cohorts, results might be less susceptible to selection bias and more easily extrapolated to a broader population. However, studies of patients with neurodegenerative disease shed light on the impact of OH on CSVD in these often more vulnerable groups, provided reference subjects are adequately chosen and analyses adjusted for important confounders. In this systematic review, we observed no clear difference in the reported association between study populations.

Second, study design and statistical methods differed between included studies. Most studies had a cross-sectional study design and showed both significant and non-significant associations. There were two studies with a longitudinal design, which give more insight into causal relation than cross-sectional studies. Importantly, both studies found no association between OH and development or progression of white matter hyperintensities.^25,26^ However, this could also be due to attrition (which ranged from 28 to 41%), in which patients with more severe progression may be more often lost to follow-up.

The association between OH an CSVD was studied with different statistical methods: t-test, linear, logistic or cox-regression models. We did find that most studies that adjusted their models for the confounding effect of SBP, did not find an association between OH and CSVD.^25,28,35^ Both OH and the occurrence of CSVD are strongly associated with uncontrolled hypertension and high SBP.^7,13^ Therefore, the OH – CSVD association might be the consequence of high SBP rather than OH. Two studies also presented a significant association between SBP and WMH burden, independent of OH status.^29,33^ These findings highlight the importance of taking SBP into account when examining the association between OH and CSVD. Although current evidence indicates hypertension treatment can reduce OH, it is frequently withheld from patients with OH due to anticipated risks of exacerbating OH and increased risk of falls.^15^ As high SBP may be a significant, if not driving, factor in the link between OH and CSVD, adequate treatment of hypertension in patients with vascular determined OH may well be pivotal to avoid clinical consequences of CSVD.

Further, there was a large variability in OH measurement and measurement of CSVD. With respect to the OH measurements most studies used active standing blood pressure measurement after seated or supine rest, whilst others used blood pressure measurement during a tilt table test; no differences in the OH-CSVD relation were observed between these studies. Further, the length of standing up/remaining upright after repeating the blood pressure measurement for OH measurement varied. If OH is causally related to CSVD, it could be expected that longer periods of OH during standing might increase the risk of CSVD. This is in line with the finding that most, but not all, studies focusing on OH at three minutes did find an association. However, prolonged blood pressure drops are also indicative of autonomic failure, which may be a consequence rather than a cause of brain injury. Future studies would benefit from use of uniform OH measurement methods, and consistent reporting of relevant OH patterns. In our opinion, most accurate information is gained when OH is measured after supine rest of at least 3 minutes, followed by BP measurements continuously after postural change, or at least after 1, 3 and 5 minutes of active standing. This method is most reflective of real life situations, and gives insight in early, prolonged an delayed OH. Measurement of the physiological impact of OH on the brain may benefit too from concurrent flow measurement using for example near infrared spectroscopy or transcranial Doppler.

Lastly, CSVD was measured with different tools. For white matter hyperintensities visual scores, e.g. Fazekas score or Scheltens score, were the most common measurement methods. However, a quantitative measure, such as white matter volume, might be more sensitive to detect subtle changes and is preferable in research settings. Similarly, taking location and number of microbleeds into account might be useful to distinguish different pathophysiological pathways, with large numbers or primarily lobar localization being more suspicious for cerebral amyloid angiopathy, and deep/infratentorial microbleeds being more common in hypertension related damage.^44,45^

In summary, this systematic review found no conclusive evidence for an association between OH and CSVD. Current evidence on the association between OH and CSVD originates mostly from cross-sectional studies, providing inconclusive and inconsistent results, two longitudinal studies found no association. High supine blood pressure might be the most important pathophysiological link between OH and CSVD. Future longitudinal studies are warranted, using standardized and fine-grained assessment of OH and CSVD, with adequate adjustment for supine blood pressure.

## Supplemental Material

sj-pdf-1-jcb-10.1177_0271678X241283226 - Supplemental material for Orthostatic hypotension and cerebral small vessel disease: A systematic review Supplemental material, sj-pdf-1-jcb-10.1177_0271678X241283226 for Orthostatic hypotension and cerebral small vessel disease: A systematic review by Julia HI Wiersinga, Frank J Wolters, Mike JL Peters, Hanneke FM Rhodius-Meester, Marijke C Trappenburg and Majon Muller in Journal of Cerebral Blood Flow & Metabolism
