# Supplementary material for: Orthostatic hypotension and cerebral small vessel disease: A systematic review
Source: J Cereb Blood Flow Metab. 2024 Sep 16;45(1):13–31. doi: 10.1177/0271678X241283226 (PMC11563541; doi:10.1177/0271678X241283226)
Supplement: sj-pdf-1-jcb-10.1177_0271678X241283226 - Supplemental material for Orthostatic hypotension and cerebral small vessel disease: A systematic review [file sj-pdf-1-jcb-10.1177_0271678X241283226.pdf]

Supplementary Material

Appendix A – Search strategy

PubMed Session Results (01 Dec 2023)

Search	Query	Items found
#3	**#1 AND #2**	945
#2	**"Cerebral Small Vessel Diseases"[Mesh] OR "small vessel*"[tiab] OR "cerebral vascular dis*"[tiab] OR "cerebrovascular dis*"[tiab] OR "cerebro-vascular dis*"[tiab] OR "cerebral vascular dam*"[tiab] OR "cerebrovascular dam*"[tiab] OR "cerebro-vascular dam*"[tiab] OR "white matter hyperintensit*"[tiab] OR "white matter hyper-intensit*"[tiab] OR "WMH"[tiab] OR "fazekas"[tiab] OR "microbleed*"[tiab] OR "micro-bleed*"[tiab] OR "lacune*"[tiab]**	62,310
#1	**"Hypotension, Orthostatic"[Mesh] OR "orthostatic hypoten*"[tiab] OR "postural hypoten*"[tiab] OR "postural intoleran*"[tiab] OR "orthostatic intoleran*"[tiab] OR ((fall[tiab] OR decreas*[tiab] OR dimin*[tiab] OR dipp*[tiab] OR orthostat*[tiab]) AND ("Blood Pressure"[Mesh] OR "blood pressure"[tiab])) OR "postural orthostat*"[tiab] OR "orthostatic collapse*"[tiab] OR "orthostatic insufficien*"[tiab] OR "orthostatic syndrome*"[tiab] OR "orthostatic symptom*"[tiab] OR "orthostatic stress*"[tiab] OR "sudden hypoten*"[tiab]**	139,271

Embase.com Session Results (01 Dec 2023)

Search	Query	Items found
#3	**#1 AND #2**	2,614
#2	**'cerebrovascular disease'/de OR 'white matter hyperintensity'/exp OR 'small vessel*':ab,ti,kw OR 'cerebral vascular dis*':ab,ti,kw OR 'cerebrovascular dis*':ab,ti,kw OR 'cerebro-vascular dis*':ab,ti,kw OR 'cerebral vascular dam*':ab,ti,kw OR 'cerebrovascular dam*':ab,ti,kw OR 'cerebro-vascular dam*':ab,ti,kw OR 'white matter hyperintensit*':ab,ti,kw OR 'white matter hyper-intensit*':ab,ti,kw OR 'WMH':ab,ti,kw OR 'fazekas':ab,ti,kw OR 'microbleed*':ab,ti,kw OR 'micro-bleed*':ab,ti,kw OR 'lacune*':ab,ti,kw**	131,838
#1	**'orthostatic hypotension'/exp OR 'orthostatic hypoten*':ab,ti,kw OR 'postural hypoten*':ab,ti,kw OR 'postural intoleran*':ab,ti,kw OR 'orthostatic intoleran*':ab,ti,kw OR ((fall:ab,ti,kw OR decreas*:ab,ti,kw OR dimin*:ab,ti,kw OR dipp*:ab,ti,kw OR orthostat*:ab,ti,kw) AND ('blood pressure'/exp OR 'blood pressure':ab,ti,kw)) OR 'postural orthostat*':ab,ti,kw OR 'orthostatic collapse*':ab,ti,kw OR 'orthostatic insufficien*':ab,ti,kw OR 'orthostatic syndrome*':ab,ti,kw OR 'orthostatic symptom*':ab,ti,kw OR 'orthostatic stress*':ab,ti,kw OR 'sudden hypoten*':ab,ti,kw**	239,245

Web of Science (Core Collection) Session Results (01 Dec 2023)

Search	Query	Items found
#3	**#1 AND #2**	1,157
#2	**TS=("small vessel*" OR "cerebral vascular dis*" OR "cerebrovascular dis*" OR**	59,025

Search	Query	Items found
	**"cerebro-vascular dis*" OR "cerebral vascular dam*" OR "cerebrovascular dam*" OR "cerebro-vascular dam*" OR "white matter hyperintensit*" OR "white matter hyper-intensit*" OR "WMH" OR "fazekas" OR "microbleed*" OR "micro-bleed*" OR "lacune*")**	
#1	**TS=("orthostatic hypoten*" OR "postural hypoten*" OR "postural intoleran*" OR "orthostatic intoleran*" OR ((fall OR decreas* OR dimin* OR dipp* OR orthostat*) AND ("blood pressure")) OR "postural orthostat*" OR "orthostatic collapse*" OR "orthostatic insufficien*" OR "orthostatic syndrome*" OR "orthostatic symptom*" OR "orthostatic stress*" OR "sudden hypoten*")**	100,326

Figure 1 – Summary of findings and risk of bias

A. Orthostatic hypotension and white matter hyperintensities

Author, year	Study design	Participants	Method	Risk of bias	Association
**Buckley et al, 2020**	Cross-sectional	Older adults	**OH**: Active standing<br>**WMH**: Scheltens score	-	✓
**Cui et al, 2020**	Cross-sectional	Older adults	**OH:** Home measured OH<br>**WMH:** WMH volume	X	✓
**Foster-Dingley et al, 2018**	Cross-sectional	Older adults	**OH:** Active standing<br>**WMH**: WMH volume	X	X
**Huang et al, 2022**	Cross-sectional	Patients with AIS	**OH**: Active standing.<br>**WMH:** Fazekas scale	+	✓
**Ten Harmsen et al, 2017**	Cross-sectional	PD patients	**OH**: Active standing<br>**WMH:** Fazekas scale	X	✓
**Jacob et al, 2022**	Cross-sectional and longitudinal	Older adults	**OH**: Active standing<br>**WMH**: WMH volume	-	X
**Juraschek et al, 2020**	Cross-sectional and longitudinal	Older adults	**OH**: Active standing<br>**WMH:** White matter grade.	X	X
**Kario et al, 2002**	Cross-sectional	Older hypertensive patients	**OH**: Tilt table test,<br>**WMH**: Advanced deep WM lesion	X	X
**Oh et al., 2013**	Cross-sectional	PD patients	**OH**: Tilt table test<br>**WMH**: Scheltens Score	X	✓
**Pilleri et al., 2012**	Cross-sectional	PD patients and matched controls	**OH**: Tilt table test<br>**WMH**: Scheltens Score	X	X
**Pilotto et al., 2021**	Cross-sectional	PD and DLB patients	**OH**: Active standing<br>**WMH**: Scheltens scale	X	X
**Shin et al, 2021**	Cross-sectional	PD patients	**OH:** Tilt table test<br>**WMH:** Fazekas scale	-	X
**Soennesyn et al, 2012**	Cross-sectional	MCI patients	**OH:** Active standing<br>**WMH:** Scheltens scale, WMH volume	-	X
**Wiersinga et al, 2023**	Cross sectional	Memory clinic patients	**OH:** Active standing<br>**WMH:** Fazekas score	-	X
**Zimmerman et al., 2020**	Cross sectional	Older patients	**OH:** Active standing<br>**WMH:** Fazekas score	X	X

Abbreviations: AIS; acute ischemic stroke, DLB; dementia with lewy bodies, MCI; mild cognitive impairment, OH; orthostatic hypotension, PD; Parkinson disease, WMH; white matter hyperintensities

Risk of bias assessment:

- X High
- \- Moderate
- \+ Low

Association

- ✓ Association
- X No Association

B. Orthostatic hypotension and lacunes

Author, year	Study design	Participants	Method	Risk of bias	Association
**Cui et al, 2020**	Cross-sectional	Older adults	**OH:** Home measured OH<br>**Lacunes:** Presence of lacunes (≥ 1)	X	✓
**Foster-Dingley et al, 2018**	Cross-sectional	MCI patients	**OH:** Active standing<br>**Lacunes**: Presence of lacunes (≥ 1)	X	X
**Jacob et al, 2022**	Cross-sectional	Older adults	**OH**: Active standing<br>**Lacunes:** Presence of lacunes (≥ 1)	-	X
**Wiersinga et al, 2023**	Cross sectional	Memory clinic patients	**OH**: Active standing<br>**Lacunes:** Presence of lacunes (≥ 1)	-	X

Abbreviations: MCI; mild cognitive impairment, OH; orthostatic hypotension

Risk of bias assessment:

X High
- Moderate
+ Low

Association

✓ Association
X No Association

C. Orthostatic hypotension and microbleeds

Author, year	Study design	Participants	Method	Risk of bias	Association
**Cui et al, 2020**	Cross-sectional	Older adults	**OH:** Home measured OH<br>**Microbleeds**: Microbleeds ≥1	X	✓
**Daida et al, 2018**	Cross-sectional	PD patients	**OH:** Active standing<br>**Microbleeds**: Microbleeds ≥ 1	X	✓
**Foster-Dingley et al, 2018**	Cross-sectional	MCI patients	**OH:** Active standing<br>**Microbleeds**: Microbleeds ≥ 1	X	X
**Jacob et al, 2022**	Cross-sectional	Older adults	**OH**: Active standing<br>**Microbleeds**: Microbleeds	-	✓
**Wiersinga et al, 2023**	Cross sectional	Memory clinic patients	**OH**: Active standing<br>**Microbleeds:** Microbleeds ≥3	-	X
**Yamashiro et al., 2015**	Cross-sectional	PD patients	**OH:** Active standing<br>**Microbleeds**: Microbleeds ≥ 1	X	X
**Yamashiro et al., 2018**	Cross-sectional	PD patients	**OH:** Active standing<br>**Microbleeds**: Microbleeds ≥ 1	+	X

Abbreviations: MCI; mild cognitive impairment, OH; orthostatic hypotension, PD; Parkinson disease

Risk of bias assessment:

- X High
- - Moderate
- + Low

Association

- ✓ Association
- X No Association

Supplemental table 1 - Modified JBI critical appraisal checklist for analytical cross sectional studies

**Questions JBI Risk of bias checklist (modified)**	**Answer options**
D1. Were the criteria for inclusion in the sample clearly defined?<br>*Yes: Inclusion criteria clearly defined, consecutive enrollment, preferably community-based samples. In patient based samples, inclusion of patients along the disease axis.*<br>*No: Inclusion criteria are not specified. Excluding patients without valid explanation and/or added value for the study (for example patients with cardiovascular disease, patients with neurogenic OH).*<br>*Unclear: not applicable.*	Yes/no/unclear
D2. Were the study subjects and the setting described in detail?<br>Yes*: Clear study setting: Description of the recruitment site of the population (clinic-, outpatient clinic-, community- based), inclusion period, inclusion center, inclusion method.*<br>*In longitudinal research: Setting of follow-up moment (follow-up time frame, reach out procedure, data collection method).*<br>No*: Study subjects and setting not sufficiently specified.*<br>Unclear: *not applicable.*	Yes/no/unclear
D3. Was the exposure measured in a valid and reliable way?<br>Yes*: OH measured using active standing or tilt table test, after supine or seated rest of at least three minutes.*<br>No*: OH measurement method unclear, not sufficient rest period before measurement.*<br>Unclear*: Measurement unclear.*	Yes/no/unclear
D4. Were objective, standard criteria used for measurement of the condition?<br>Yes*: OH using consensus definition, measured within 3 minutes of standing up/tilting to at least 60* ^{0}*C.*<br>No*: Not using consensus definition: for example OH measurement later than three minutes, diagnosis based on complaints.*<br>Unclear: *OH criteria unclear.*	Yes/no/unclear
D5. Were confounding factors identified?<br>Yes*: Identification of following confounding factors: age, sex, systolic blood pressure, OH-inducing medication and to lesser extent cardiovascular disease, diabetes and, neurological disease (PD/DLB).*<br>No*: Important confounders not identified and/or presented.*<br>Unclear*: Unclear if confounding factors are identified.*	Yes/no/unclear
D6. Were strategies to deal with confounding factors stated?<br>Yes*: Analyses adjusted for important confounding factors (see above).*<br>No*: Unadjusted analyses.*<br>Unclear*: Adjustment unclear.*	Yes/no/unclear
D7. Were the outcomes measured in a valid and reliable way?<br>Yes*: CSVD measured with MRI or CT, using validated measurement tools, assessment by trained professional(s) blinded for OH status.*<br>No*: Unvalidated measurement tool, professional(s) not blinded.*<br>Unclear*: Assessment unclear.*	Yes/no/unclear
D8. Was appropriate statistical analysis used?	Yes/no/unclear

Yes*: Statistical analysis of association includes logistic/linear regression analysis or ANCOVA, adjusted for important confounding factors (see above).*<br>*Cross-sectional data: Missing data <5%, otherwise imputed.*<br>*For longitudinal studies: Information on re-examination, loss to follow-up <=20%.*<br>No: *Percentage and t-test, other. Cross-sectional missing data >5% and not imputed or overall >25%. Longitudinal missing data > 20%.*<br>Unclear*: Statistical analysis unclear.*	

Abbreviations: ANCOVA; Analysis of covariance, CSVD; cerebral small vessel disease, CT; computer tomography, DLB; Dementia with Lewy bodies, JBI; Johanna Briggs institute MRI; magnetic resonance imaging, OH: orthostatic hypotension, PD; Parkinson disease
